# The impact of side effects from outpatient chemotherapy on presenteeism in breast cancer patients: a prospective analysis

**DOI:** 10.1186/s40064-016-1979-x

**Published:** 2016-03-15

**Authors:** Tomoya Tachi, Hitomi Teramachi, Kazuhide Tanaka, Shoko Asano, Tomohiro Osawa, Azusa Kawashima, Akiyo Hori, Masahiro Yasuda, Takashi Mizui, Takumi Nakada, Yoshihiro Noguchi, Teruo Tsuchiya, Chitoshi Goto

**Affiliations:** Laboratory of Clinical Pharmacy, Gifu Pharmaceutical University, 1-25-4, Daigakunishi, Gifu-shi, Gifu, 501-1196 Japan; Department of Pharmacy, Gifu Municipal Hospital, 7-1 Kashima-cho, Gifu-shi, Gifu, 500-8513 Japan; Department of Breast Surgery, Gifu Municipal Hospital, 7-1 Kashima-cho, Gifu-shi, Gifu, 500-8513 Japan; Community Health Support and Research Center, 15 Takehana-cho Nishikimachi, Hashima-shi, Gifu, 501-6242 Japan

**Keywords:** Presenteeism, Absenteeism, Outpatient chemotherapy, Breast cancer

## Abstract

In the field of occupational health services, productivity loss can be expressed by absenteeism (i.e., employees being absent from work and taking leave due to health problems) and presenteeism (i.e., a reduction in the ability to perform one’s tasks at work). Similar to absenteeism, it is important to assess presenteeism because it can severely reduce productivity. Despite numerous reports about the impact of disease and medical treatments on presenteeism, there is a lack of data regarding the influence of medication side effects. In this study, a prospective analysis was conducted via questionnaire survey to clarify the influence of the side effects of anticancer drugs on presenteeism in workers receiving outpatient chemotherapy for breast cancer. Between December 2012 and November 2013, the influence of side effects on the quality of life, absenteeism, and presenteeism was investigated via a questionnaire conducted before and after 1 course of chemotherapy in 19 currently employed breast cancer patients receiving outpatient chemotherapy for the first time at Gifu Municipal Hospital, Japan. The rate of absenteeism was 24.7 %, resulting in financial losses of 2002 yen/day (national statistical data) and 881 yen/day (our questionnaire data). The rate of presenteeism was 33.7 %, resulting in financial losses of 1354 yen/day (national statistical data) and 1263 yen/day (our questionnaire data). Furthermore, a significant positive correlation was observed between absenteeism and presenteeism (*r* = 0.687, *p* = 0.001), suggesting that the productivity losses associated with presenteeism due to the side effects of anticancer drugs in breast cancer patients are large and similar to that associated with absenteeism in these patients. Our results may be useful for improving the occupational health of workers receiving chemotherapy for cancer.

## Background

Decreases in labor productivity due to health problems occur not only from an employee being absent from work or taking leave (absenteeism), but also from reduced productivity whilst at work (presenteeism), and this presents an important problem facing the field of occupational health. Presenteeism is defined as a reduced ability to perform at work due to health problems (Burton et al. 1999). The losses businesses incur due to presenteeism have been reported to eclipse the losses due to absenteeism (Loeppke et al. 2003, 2007; Goetzel et al. 2004). Therefore, investigation into the impact of specific health problems on presenteeism is very important for the creation of measures in the field of occupational health. For example, data attained from surveys on presenteeism is very beneficial for government and businesses for planning and assessing occupational health plans, and calculating the cost-effectiveness of the utilization of occupational health activities. However, although numerous reports on presenteeism arising from disease and treatment exist throughout the world (Koopmanschap et al. 2005; Schultz and Edington 2007; Pauly et al. 2008; Schultz et al. 2009; Bockerman and Laukkanen 2010; Brown et al. 2011; Cocker et al. 2011), there have been very few investigative reports conducted in Japan (Yamashita and Arakida 2006; Wada et al. 2007; Minami et al. 2010; Wada et al. 2013). Presenteeism is considered to be affected by working environments and work–life balance (Dew et al. 2005); thus, given that these differ according to country and culture, the accumulation of data on presenteeism in Japan is considered to be essential to the field of occupational health in Japan.

There are numerous reports on the beneficial effects of pharmaceuticals on presenteeism (Schultz et al. 2009); however, there are no investigative reports that focus on the impact of the side effects of pharmaceuticals on presenteeism. In recent years, in accordance with advances in healthcare cost containment and home healthcare, the administration of anticancer drugs has transitioned from inpatient care to outpatient clinics, and outpatient chemotherapy is now widely practiced. The side effects associated with anticancer drugs affect the quality of life (QOL) of cancer patients (Fallowfield et al. 2006; Taira et al. 2014; Tachi et al. 2015). We hypothesize that, in addition to a reduction in QOL, presenteeism is increased in patients receiving outpatient chemotherapy. Thus, presenteeism due to the side effects of chemotherapy could potentially become a significant problem for the occupational health field.

The incidence of breast cancer has been increasing. The majority of cases occur in women in their late 50s, but there has been an increasing number of cases in women in their late 20s (National Cancer Center 2001; National Cancer Institute 2014). With a comparatively younger generation of women affected by breast cancer, a growing number of patients receive outpatient chemotherapy while continuing their employment. In a previous report, we demonstrated that the side effects of anticancer drugs decrease the QOL of breast cancer patients receiving outpatient chemotherapy (Tachi et al. 2015; Tanaka et al. 2015). We hypothesize that, due to the side effects, QOL decreases and presenteeism increases for workers receiving outpatient chemotherapy.

The objective of this prospective study was to clarify, via questionnaire, the impact of the side effects of anticancer drugs on presenteeism in workers receiving outpatient chemotherapy for breast cancer.

## Methods

### Subjects

All currently employed breast cancer patients who received outpatient chemotherapy for the first time at Gifu Municipal Hospital, Japan, between December 2012 and November 2013 were enrolled in this study. Outpatient chemotherapy for breast cancer patients is changing rapidly because novel anti-cancer drugs, regimens, and supportive therapies have been developed. Furthermore, chemotherapy has shifted from inpatient to outpatient settings due to the medical cost reduction induced by the Japanese government. In order to reduce the influence of this shift and focus on current outpatient chemotherapy in Japan, we selected the limited study period of 1 year over the sample size.

### Survey method

The survey questionnaire was conducted before and after a single course of outpatient chemotherapy.

The pre-treatment questionnaire consisted of the Japanese edition of the EuroQol 5 Dimension (EQ-5D) (EuroQol Group 1990; Okamoto et al. 2004), the Quality of Life Questionnaire for Cancer Patients Treated with Anticancer Drugs (QOL-ACD) (Shimozuma et al. 1994; Kurihara et al. 1999), and items regarding patient attributes, such as marital status, whether they lived alone or with others and the number of people in their household, current employment, employment before diagnosis, education, income, and whether they were health personnel.

**Fig. 1:**
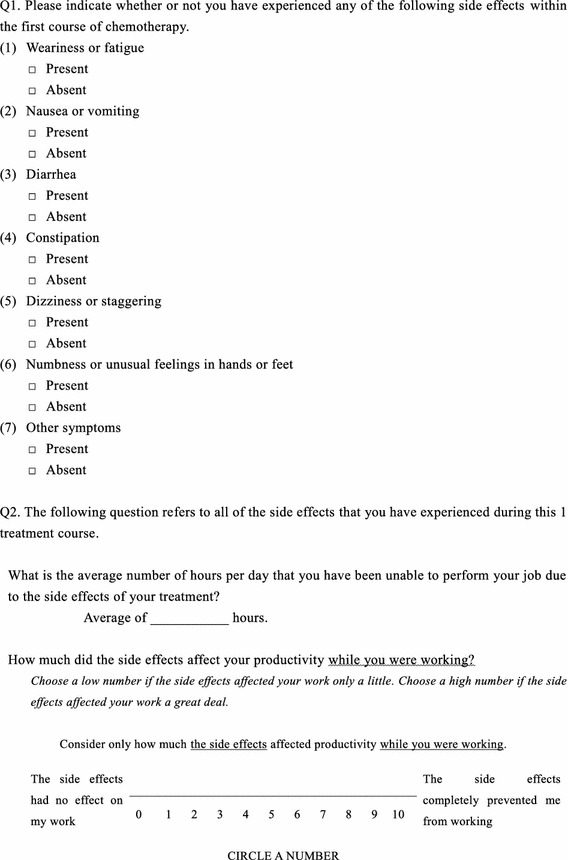
Content of the post-treatment questionnaire

The post-treatment questionnaire consisted of the EQ-5D, the QOL-ACD, and items concerning the side effects of anticancer drugs, absenteeism, and presenteeism (Fig. 1). The questions concerning presenteeism were based on the Work Productivity and Activity Impairment Questionnaire General Health v2.0 (WPAI-GH) (Reilly et al. 1993). In our study, questionnaire research was performed after 1 course of chemotherapy during which we were able to obtain comprehensive information regarding the conditions.

In addition, we collected data regarding the patients’ age, TNM breast cancer staging, performance status (PS), human epidermal growth factor receptor type 2 (HER2), estrogen receptor (ER), progesterone receptor (PgR), the purpose of anticancer drug treatment, and the treatment regimen from electronic medical records.

### Analytical methods and statistical processing

QOL was evaluated with the utility value and ratio of persons with problems for each of the 5 items calculated from the EQ-5D, and the total score of all items and the average score of each subscale obtained from the QOL-ACD.

For evaluating absenteeism and presenteeism arising from anticancer drug treatment, we used national statistical data from the 2012 Basic Survey on Wage Structure (i.e., daily working time and wages for regular and non-regular staff by age) published by the Ministry of Health, Labour and Welfare of Japan (2012), and data from our questionnaire (i.e., daily wage calculated from the average annual income based on the questionnaire results) (August 21, 2015: 1 USD = 122.5 yen, 1 euro = 138.6 yen].

#### Absenteeism

$${\text{Rate}}\;{\text{of}}\;{\text{labor}}\;{\text{loss}}\;\left( \% \right) = {\text{daily}}\;{\text{hours}}\;{\text{lost}}\,\left( {\text{h}} \right)/{\text{daily}}\;{\text{working}}\;{\text{time}}\,\left[ {\text{national statistical data}} \right]\left( {\text{h}} \right)$$

#### Presenteeism

$${\text{Rate}}\;{\text{of}}\;{\text{labor}}\;{\text{loss}}\,\left( \% \right) = {\text{questionnaire}}\;{\text{value}}\;\left( {{\text{range}},0{-}10} \right) \times 10$$

#### Amount of labor loss (for absenteeism or presenteeism)

$${\text{Amount}}\;{\text{of}}\;{\text{labor}}\;{\text{loss}}\,\left[ {{\text{national}}\;{\text{statistical}}\;{\text{data}}} \right]\left( {\text{yen/day}} \right) = {\text{daily}}\;{\text{wage}}\,\left[ {{\text{national}}\;{\text{statistical}}\;{\text{data}}} \right]\left( {{\text{yen}}/{\text{day}}} \right) \times {\text{rate}}\;{\text{of}}\;{\text{labor}}\;{\text{loss}}\,\left( \% \right)/100$$

$${\text{Amount}}\;{\text{of}}\;{\text{labor}}\;{\text{loss }}\,\left[ {{\text{our}}\;{\text{questionnaire}}\;{\text{data}}} \right]\left( {\text{yen/day}} \right) = {\text{daily}}\;{\text{wage}}\left[ {{\text{our}}\;{\text{questionnaire}}\;{\text{data}}} \right]\left( {\text{yen/day}} \right) \times {\text{rate}}\;{\text{of}}\;{\text{labor}}\;{\text{loss}}\,\left( \% \right)/100$$

Daily working time calculated from the national statistical data was used for evaluating absenteeism, as substitute for the sum of working time obtained from a questionnaire because of variations in the working days per week for people in Japan.

Furthermore, to account for the losses associated with both absenteeism and presenteeism, we summed the losses due to absenteeism and the losses due to presenteeism after deducting the losses through absenteeism.

SPSS 22 software (IBM, Armonk, NY, USA) was used for statistical analysis. A paired *t* test was used to test the difference between the total score from the QOL-ACD and average score for each subscale, and the utility value before and after 1 treatment course. Fisher’s exact test was used for the between-group comparisons to evaluate the effect of the presence or absence of each side effect on the ratio of patients whose condition had deteriorated before and after treatment according to the 5 items in the EQ-5D. The Mann–Whitney U test was used to evaluate the differences in the changes in the QOL, absenteeism, and presenteeism between before and after chemotherapy stratified by the presence or absence of each side effect. Spearman’s rank correlation analysis was used to evaluate the correlations between absenteeism or presenteeism, and QOL. A value of *p* < 0.05 was considered statistically significant.

### Ethics

This study was conducted in accordance with the Declaration of Helsinki and other Japanese research guidelines. The study protocol was approved by the ethics committee of Gifu Pharmaceutical University and Gifu Municipal Hospital (Bioethics Committee of Gifu Pharmaceutical University, Research Ethics Committee No. 128, respectively). The study documents were explained to the patients and letters of informed consent were acquired prior to enrolment. Furthermore, the patients participated in the study voluntarily and were permitted to withdraw from the study at any point regardless of the reason.

## Results

### Patient characteristics

All enrolled patients (n = 19) answered the questionnaire twice and no patients declined to participate in the survey. The patient characteristics are shown in Table 1. The patients were all women with a mean age of 55.6 years. Of the 19 patients, 36.8 % had stage 1 breast cancer, and 100 % had a PS value of 0. Seven patients (36.8 %) were HER2-(3+), 13 (68.4 %) were ER-positive, and 12 (63.2 %) were PgR-positive. The most widely used treatment regimen was EC (epirubicin/cyclophosphamide) chemotherapy using epirubicin and cyclophosphamide (42.1 % of patients). Furthermore, 73.7 % of the patients were married and 100 % lived with at least one other person in their household. All participants were currently employed, and their employment status had not changed from before diagnosis. The majority of patients worked part-time or had temporary employment (52.6 %), 31.6 % of patients were employed full-time or self-employed, and 15.8 % of patients did not fit into either category.

**Table 1:** Patient attributes

	Mean ± SD	
Age (year)	55.6 ± 9.7	

	*n*	%
Stage		
1	7	36.8
2	6	31.6
3	4	21.1
4	2	10.5
TNM classification		
T1, N0, M0	8	42.1
T2, N0, M0	3	15.8
T2, N1, M0	2	10.5
T4, N0, M0	2	10.5
T4, N2, M0	2	10.5
T4, N0, M1	2	10.5
PS		
0	19	100.0
HER2		
3+	7	36.8
2+	1	5.3
1+	7	36.8
(−)	4	21.1
ER		
(+)	13	68.4
(−)	3	15.8
(±)	3	15.8
PgR		
(+)	12	63.2
(−)	3	15.8
(±)	4	21.1
Purpose of chemotherapy		
Adjuvant	7	36.8
Neoadjuvant	11	57.9
Symptom relief	1	5.3
Regimen		
EC (every 3w)	8	42.1
TRZ + nabPTX (every 3w)	3	15.8
TC (every 3w)	2	10.5
3w-nabPTX (every 3w)	3	15.8
LPR (every 4w)	3	15.8
Marital status		
Married	14	73.7
Unmarried	5	26.3
Cohabitants		
Yes	19	100.0
No	0	0.0
Current occupation		
Temporary employment, part-time employment	10	52.6
Company employee, self-employed	6	31.6
Public official	0	0.0
Other	3	15.8
Current annual income		
0–999,999 yen	10	52.6
1,000,000–4,999,999 yen	7	36.8
5,000,000–9,999,999 yen	2	10.5
10,000,000– yen	0	0.0
Occupation before diagnosis		
Temporary employment, part-time employment	10	52.6
Company employee, self-employed	6	31.6
Public official	0	0.0
Other	3	15.8
Education		
Junior school graduate	3	15.8
High school graduate	7	36.8
Vocational school, junior college, technical school graduate	8	42.1
University graduate, postgraduate	1	5.3
Experience as a healthcare professional		
Currently a healthcare professional	0	0.0
Was a healthcare professional in the past	1	5.3
Was not a healthcare professional in the past	18	94.7

*SD* standard deviation, *PS* performance status, *HER2* human epidermal growth factor receptor type 2, *ER* estrogen receptor, *PgR* progesterone receptor, *EC* epirubicin/cyclophosphamide, *TC* docetaxel/cyclophosphamide, *TRZ* trastuzumab, *LPR* leuprorelin, *nabPTX* nab-paclitaxel, *3w* 3 weeks, *4w* 4 weeks

### QOL assessment

The QOL as assessed by the EQ-5D before and after 1 treatment course is shown in Table 2. The utility value decreased significantly after treatment (*p* < 0.001). Specifically, there was a significant increase in the number of patients who experienced problems with usual activities after treatment (*p* = 0.020).

**Table 2:** The EQ-5D utility value and 5 dimensions before and after 1 course of outpatient chemotherapy

	Before CT	After CT	*p*
Utility value	0.907 ± 0.128	0.771 ± 0.125	<0.001*
Dimensions	Some or major problems (%)		
Mobility	5.3	15.8	0.604
Personal care	0	0	1.000
Usual activities	0	31.6	0.020*
Pain/discomfort	21.1	42.1	0.295
Anxiety/depression	26.3	57.9	0.099

*CT* chemotherapy

*p < 0.05

The QOL as assessed by the QOL-ACD before and after 1 treatment course is shown in Table 3. The total score for the QOL-ACD decreased significantly after treatment (*p* = 0.045). For the QOL-ACD subscales, the mean scores for activity and physical condition decreased significantly after treatment (*p* = 0.021 and *p* = 0.003, respectively); however, a significant increase in the mean score was observed for social relationships (*p* = 0.010).

**Table 3:** The QOL-ACD total score and mean score for each subscale before and after 1 course of outpatient chemotherapy

	Before CT	After CT	*p*
Total score	93.6 ± 6.9	89.1 ± 9.9	0.045*
Average score of subscales			
Activity	4.83 ± 0.32	4.45 ± 0.77	0.021*
Physical condition	4.60 ± 0.27	4.11 ± 0.56	0.003*
Psychological condition	4.28 ± 0.52	3.98 ± 0.66	0.077
Social relationships	2.43 ± 0.65	2.81 ± 0.70	0.010*

*CT* chemotherapy, mean ± standard deviation

*p < 0.05

### Subjective side effects

The following side effects were reported by the patients: weariness or fatigue (68.4 %), nausea or vomiting (31.6 %), diarrhea (10.5 %), constipation (47.4 %), dizziness or staggering (21.1 %), numbness or unusual feelings in hands or feet (42.1 %), and other side effects (47.4 %).

### Assessment of absenteeism and presenteeism

The losses in productivity and costs due to absenteeism and presenteeism are shown in Table 4. The rate of absenteeism was 24.7 %, resulting in 1.79 h/day of lost working hours and a financial loss of 2002 and 881 yen/day according to national statistical and our questionnaire data, respectively. The rate of presenteeism was 33.7 %, resulting in financial losses of 1354 and 1263 yen/day according to national statistical and our questionnaire data, respectively. Furthermore, when absenteeism and presenteeism were assessed together, the total cost of productivity loss was 3356 and 2144 yen/day according to national statistical and our questionnaire data, respectively.

**Table 4:** Absenteeism and presenteeism

	Mean ± SD (unit)
Absenteeism	
Loss of labor time	1.79 ± 2.92 (h/day)
Rate of labor loss	24.7 ± 39.1 (%)
Amount of labor loss	
Calculated according to national statistical data	2002 ± 3374 (yen/day)
Calculated according to the questionnaire data	881 ± 1883 (yen/day)
Presenteeism	
Rate of labor loss	33.7 ± 33.7 (%)
Amount of labor loss	
Calculated according to national statistical data	1354 ± 1907 (yen/day)
Calculated according to the questionnaire data	1263 ± 1960 (yen/day)
Absenteeism + Presenteeism	
Amount of labor loss	
Calculated according to national statistical data	3356 ± 3796 (yen/day)
Calculated according to the questionnaire data	2144 ± 2623 (yen/day)

*SD* standard deviation

The correlation between absenteeism or presenteeism and QOL is shown in Table 5. A significant positive correlation was observed between absenteeism and presenteeism (*r* = 0.687, *p* = 0.001). Furthermore, significant negative correlations were observed in post-treatment changes between the QOL-ACD scores and absenteeism or presenteeism (absenteeism: *r* = −0.528, *p* = 0.020; presenteeism: *r* = −0.625, *p* = 0.004). No significant correlations were observed between absenteeism or presenteeism and post-treatment changes in the EQ-5D scores.

**Table 5:** Correlations among absenteeism, presenteeism and quality of life

Adverse events	Correlation coefficient^#^	*p*
Absenteeism versus Presenteeism	0.687	0.001*
Absenteeism versus ΔEQ-5D	0.273	0.258
Absenteeism versus ΔQOL-ACD	−0.528	0.020*
Presenteeism versus ΔEQ-5D	0.169	0.489
Presenteeism versus ΔQOL-ACD	−0.625	0.004*

Absenteeism and presenteeism represent respective rates of labor loss, ΔEQ-5D and ΔQOL-ACD represent respective changes in utility value before and after a course of chemotherapy, and changes in the total QOL-ACD score

^#^Spearman’s rank correlation coefficient, *p < 0.05

The correlation between absenteeism or presenteeism and the QOL based on the subscales of the QOL-ACD is shown in Table 6. A significant negative correlation was observed between absenteeism or presenteeism and post-treatment changes in the activity subscale score in the QOL-ACD (absenteeism: *r* = −0.570, *p* = 0.011; presenteeism: *r* = −0.736, *p* < 0.001); however, no significant correlations were observed for the other subscale scores.

**Table 6:** Correlations between absenteeism and presenteeism, and quality of life (subscales of QOL-ACD)

Adverse events	Correlation coefficient^#^	*p*
Absenteeism versus ΔActivity	−0.570	0.011^*^
Absenteeism versus ΔPhysical condition	−0.296	0.219
Absenteeism versus ΔPsychological condition	−0.240	0.322
Absenteeism versus ΔSocial relationships	−0.224	0.357
Presenteeism versus ΔActivity	−0.736	<0.001*
Presenteeism versus ΔPhysical condition	−0.429	0.067
Presenteeism versus ΔPsychological condition	−0.163	0.505
Presenteeism versus ΔSocial relationships	−0.184	0.450

Absenteeism and presenteeism represent respective rates of labor loss, ΔActivity, ΔPhysical condition, ΔPsychological condition and ΔSocial relationships represent respective changes in average scores of QOL-ACD subscales (Activity, Physical condition, Psychological condition and Social relationship)

^#^Spearman’s rank correlation coefficient, *p < 0.05

The changes in the QOL, absenteeism, and presenteeism between before and after chemotherapy, stratified by the presence or absence of each side effect, are shown in Table 7. Diarrhea and dizziness or staggering were not evaluated because of the small sample size of patients with the side effects. The reduction in the QOL-ACD and activity was significantly greater in patients with nausea or vomiting than in those without nausea or vomiting (respectively, *p* = 0.046 and *p* = 0.005). The changes in the other subscale scores in the QOL-ACD, absenteeism, and presenteeism were not significantly different between those with and without the other side effects.

**Table 7:** Influence of each side effect on quality of life, absenteeism and presenteeism

	Weariness			Nausea or vomiting			Constipation			Numbness or strange feelings in hands or feet		
	(+), *n* = 13	(−), *n* = 6	*p*	(+), *n* = 6	(−), *n* = 13	*p*	(+), *n* = 9	(−), *n* = 10	*p*	(+), *n* = 8	(−), *n* = 11	*p*
ΔEQ-5D	−0.124 ± 0.143	−0.162 ± 0.135	0.579	−0.135 ± 0.145	−0.137 ± 0.140	1.000	−0.114 ± 0.126	−0.156 ± 0.151	0.720	−0.174 ± 0.156	−0.109 ± 0.123	0.395
ΔQOL-ACD	−4.92 ± 10.33	−3.67 ± 0.669	0.639	−11.17 ± 7.68	−1.46 ± 8.31	0.046^*^	−4.22 ± 10.47	−4.80 ± 8.38	0.842	−8.38 ± 11.41	−1.73 ± 6.27	0.075
ΔActivity	−0.397 ± 0.699	−0.361 ± 0.653	0.831	−0.722 ± 0.502	−0.231 ± 0.692	0.087	−0.593 ± 0.913	−0.200 ± 0.258	0.549	−0.729 ± 0.766	−0.136 ± 0.476	0.051
ΔPhysical condition	−0.513 ± 0.665	−0.417 ± 0.545	0.701	−1.028 ± 0.287	−0.231 ± 0.563	0.005^*^	−0.463 ± 0.735	−0.500 ± 0.527	0.968	−0.625 ± 0.533	−0.379 ± 0.675	0.313
ΔPsychological conditition	−0.292 ± 0.831	−0.333 ± 0.393	0.521	−0.567 ± 0.898	−0.185 ± 0.608	0.368	−0.178 ± 0.682	−0.420 ± 0.751	0.497	−0.600 ± 1.000	−0.091 ± 0.288	0.395
ΔSocial relationships	0.354 ± 0.524	0.433 ± 0.720	0.701	0.400 ± 0.358	0.369 ± 0.663	0.831	0.511 ± 0.437	0.260 ± 0.674	0.400	0.525 ± 0.354	0.273 ± 0.689	0.492
Absenteeism	29.3 ± 41.0	14.6 ± 35.7	0.521	44.8 ± 49.6	15.4 ± 31.1	0.244	26.5 ± 40.0	23.0 ± 40.4	1.000	42.3 ± 45.9	11.8 ± 29.1	0.238
Presenteeism	38.5 ± 35.6	23.3 ± 29.4	0.323	53.3 ± 33.3	24.6 ± 31.0	0.058	43.3 ± 35.7	25.0 ± 31.0	0.243	51.3 ± 41.9	20.9 ± 19.7	0.152

Absenteeism and presenteeism represent respective rates of labor loss, ΔEQ-5D and ΔQOL-ACD represent respective changes in utility value before and after a course of chemotherapy, and changes in the total QOL-ACD score. ΔActivity, ΔPhysical condition, ΔPsychological condition and ΔSocial relationships represent respective changes in average scores of QOL-ACD subscales (Activity, Physical condition, Psychological condition and Social relationship). Mean ± standard deviation, *p < 0.05

## Discussion

We conducted a questionnaire-based prospective analysis to investigate the impact of the side effects of anticancer drugs on presenteeism in working patients receiving outpatient chemotherapy for breast cancer. Furthermore, we assessed the correlation between presenteeism or absenteeism due to treatment side effects and changes in QOL.

The majority of the patients in this study had stage 1 breast cancer and was ER-positive and PgR-positive, which was consistent with the statistical data in Japan (The Japanese Breast Cancer Society 2012). A high number of patients had a PS value of 0 and HER2-(3+), underwent chemotherapy as neoadjuvant therapy, and were administered the EC regimen for chemotherapy compared to the statistical data in Japan (The Japanese Breast Cancer Society 2012), which included chemotherapy patients and those who did not receive chemotherapy in an outpatient setting. The demographics of this study would be similar to the distribution of breast cancer patients receiving outpatient chemotherapy in Japan. The percentage of temporary and part-time workers was 52.6 %, which correspond to the present Japanese labor market (45.9 %) (Ministry of Health, Labour and Welfare of Japan 2011).

Significant reductions in the QOL were observed after 1 treatment course, according to the total scores of the EQ-5D and QOL-ACD. There was a significant increase in patients who reported having difficulties with usual activities in the EQ-5D. The presence of problems in some dimensions was not significantly different between before and after chemotherapy, which might be due to the small number of patients. In addition, there might be the possibility of the influence of the diagnosis on the EQ-5D, especially depression/anxiety. The average scores for activity and physical condition decreased significantly in the QOL-ACD after 1 treatment course. However, the average scores for social relationships increased significantly after 1 treatment course.

The loss of productivity due to absenteeism and presenteeism was 24.7 and 33.7 %, respectively. The estimated financial impact of lost productivity ranged 881–2002 and 1263–1354 yen/day for absenteeism and presenteeism, respectively. Similar to absenteeism, there were significant productivity losses associated with presenteeism due to the side effects of chemotherapy. It has been reported that business losses from presenteeism were greater than that from absenteeism (Loeppke et al. 2003, 2007; Goetzel et al. 2004). According to our results, productivity losses due to presenteeism were at least similar in magnitude to that of absenteeism. Furthermore, the financial impact for both absenteeism and presenteeism was 2144–3356 yen/day, amounting to a 40–50 % loss in productivity per day. Therefore, the impact of absenteeism and presenteeism due to the side effects of chemotherapy are considered strongly significant. According to these results, side effects due to chemotherapy greatly led to both absenteeism and presenteeism.

A moderate positive correlation was observed between absenteeism and presenteeism, suggesting that employees with absenteeism are also likely to be affected by presenteeism. A higher reduction in the QOL suggested a higher degree of absenteeism or presenteeism based on the moderate negative correlation observed between absenteeism or presenteeism and post-treatment changes in the QOL (QOL-ACD). There were no corresponding significant correlations observed between absenteeism or presenteeism and post-treatment changes in the EQ-5D. Differences in the designs of these QOL measures may account for these findings. The QOL-ACD was developed specifically for cancer patients and consisted of 5-stage answers to 23 questions; while the EQ-5D was designed for the general population with 3-stage answers to 5 questions. Therefore, the measurement sensitivity of the QOL in the EQ-5D in cancer patients was considered to be lower than that in the QOL-ACD. Furthermore, a higher reduction in activity suggested a higher degree in absenteeism or presenteeism based on our observation of a moderate negative correlation between absenteeism and post-treatment changes in activity (QOL-ACD) and a strong negative correlation between presenteeism and post-treatment changes in activity (QOL-ACD). Activity would be one of the factors determining the correlation between absenteeism or presenteeism and the QOL-ACD.

The reductions in the QOL-ACD and physical condition in patients with nausea or vomiting were significantly greater compared to those without side effects, suggesting that the QOL, especially their physical condition, was influenced by nausea and vomiting due to chemotherapy. Presenteeism in patients with nausea or vomiting tended to be greater compared to those without the side effect, therefore nausea or vomiting might influence presenteeism. However, the changes in the other subscale scores of the QOL-ACD, absenteeism, and presenteeism were not significant different between those with and without the other side effects. These results might occur due to limitations in sample size.

One of the limitations of our study is the small sample size (19 patients) owing to the single-site design. As explained earlier, we selected the limited period of 1 year over sample size, in order to reduce the influence of the shift in chemotherapy in Japan. The content validity of the questionnaire (the efficiency of question or questions to correctly express the content to be clarified in the study) might be another limitation in this study. In our study, presenteeism was evaluated, using an ordinal scale, by directly asking about work productivity during work as a single question. Therefore, the content validity of the questionnaire for evaluating presenteeism would be adequate in an exploratory research to investigate the impact of the side effects of anticancer drugs on presenteeism in workers receiving outpatient chemotherapy for breast cancer, especially in Japan. In the future, further investigations regarding the influence of the side effects of breast cancer chemotherapy on presenteeism should be conducted in a larger number of patients to verify the rigid content validity of the questionnaire. Moreover, our analysis was based on the acute phase side effects because we only included patients receiving chemotherapy for the first time. Given that chemotherapy is not usually completed in a single course, similar side effects can be expected with repeated sessions of chemotherapy. As the treatment continues and number of courses of chemotherapy increases, chronic phase side effects may emerge and the subsiding of acute side effects may be delayed. Therefore, it will be necessary to investigate the impact of long-term chronic and acute side effects of anticancer drugs in future studies about presenteeism. Furthermore, it will also be necessary to investigate this impact in specific subsamples (i.e., different age groups and types of chemotherapy) and to clarify the influence of the side effects of chemotherapy for other types of carcinomas, in addition to breast cancer, on presenteeism.

As outpatient chemotherapy is becoming more common, the number of people receiving anticancer drug treatment while continuing to work is increasing. Therefore, the findings from this survey regarding presenteeism of patients receiving cancer treatment are important for improving occupational health of workers receiving chemotherapy for cancer. In previous studies, presenteeism is influenced by characteristics related to workers and workplaces (Bockerman and Ilmakunnas 2008), and working time match between desired and actual weekly working hours reduces presenteeism (Bockerman and Laukkanen 2010). Earlier evidence points to the fact that special attention should be paid to working time arrangements, workers’ replacement practices, attendance pressure factors, and personal attitudes (Aronsson et al. 2000; Aronsson and Gustafsson 2005; Hansen and Andersen 2008). Therefore, the findings of our study can be helpful for planning a practical policy for workers receiving chemotherapy for cancer, such as industrial health control for workers, introduction of a leave system for workers by the government, and subsidizing companies that promote the leave system from the government.

## Conclusions

According to the findings of this prospective analysis, the side effects of anticancer drugs have a significant impact on presenteeism in workers receiving outpatient chemotherapy for breast cancer in Japan. Especially, nausea or vomiting due to chemotherapy influences the QOL (i.e., activity that might influence presenteeism); therefore supportive therapy and instruction to patients for the prevention of the side effects might be more improved. We believe that the results would be useful as an exploratory study, but further investigations should be conducted in a larger number of patients to clarify the influence of the side effects of breast cancer chemotherapy on presenteeism.

## References

[CR1] Aronsson G, Gustafsson K (2005). Sickness presenteeism: prevalence, attendance-pressure factors, and an outline of a model for research. J Occup Environ Med.

[CR2] Aronsson G, Gustafsson K, Dallner M (2000). Sick but yet at work. An empirical study of sickness presenteeism. J Epidemiol Community Health.

[CR3] Bockerman P, Ilmakunnas P (2008). Interaction of working conditions, job satisfaction, and sickness absences: evidence from a representative sample of employees. Soc Sci Med.

[CR4] Bockerman P, Laukkanen E (2010). Predictors of sickness absence and presenteeism: does the pattern differ by a respondent’s health?. J Occup Environ Med.

[CR5] Brown HE, Gilson ND, Burton NW, Brown WJ (2011). Does physical activity impact on presenteeism and other indicators of workplace well-being?. Sports Med.

[CR6] Burton WN, Conti DJ, Chen CY, Schultz AB, Edington DW (1999). The role of health risk factors and disease on worker productivity. J Occup Environ Med.

[CR7] Cocker F, Martin A, Scott J, Venn A, Otahal P, Sanderson K (2011). Factors associated with presenteeism among employed Australian adults reporting lifetime major depression with 12-month symptoms. J Affect Disord.

[CR8] Dew K, Keefe V, Small K (2005). ‘Choosing’ to work when sick: workplace presenteeism. Soc Sci Med.

[CR9] EuroQol Group (1990). EuroQol—a new facility for the measurement of health-related quality of life. Health Policy.

[CR10] Fallowfield LJ, Bliss JM, Porter LS, Price MH, Snowdon CF, Jones SE, Coombes RC, Hall E (2006). Quality of life in the intergroup exemestane study: a randomized trial of exemestane versus continued tamoxifen after 2 to 3 years of tamoxifen in postmenopausal women with primary breast cancer. J Clin Oncol.

[CR11] Goetzel RZ, Long SR, Ozminkowski RJ, Hawkins K, Wang S, Lynch W (2004). Health, absence, disability, and presenteeism cost estimates of certain physical and mental health conditions affecting U.S. employers. J Occup Environ Med.

[CR12] Hansen CD, Andersen JH (2008). Going ill to work–what personal circumstances, attitudes and work-related factors are associated with sickness presenteeism?. Soc Sci Med.

[CR13] Koopmanschap M, Burdorf A, Jacob K, Meerding WJ, Brouwer W, Severens H (2005). Measuring productivity changes in economic evaluation: setting the research agenda. Pharmacoeconomics.

[CR14] Kurihara M, Shimizu H, Tsuboi K, Kobayashi K, Murakami M, Eguchi K, Shimozuma K (1999). Development of quality of life questionnaire in Japan: quality of life assessment of cancer patients receiving chemotherapy. Psychooncology.

[CR15] Loeppke R, Hymel PA, Lofland JH, Pizzi LT, Konicki DL, Anstadt GW, Baase C, Fortuna J, Scharf T (2003). Health-related workplace productivity measurement: general and migraine-specific recommendations from the ACOEM Expert Panel. J Occup Environ Med.

[CR16] Loeppke R, Taitel M, Richling D, Parry T, Kessler RC, Hymel P, Konicki D (2007). Health and productivity as a business strategy. J Occup Environ Med.

[CR17] Minami Y, Shiozaki Y, Kato C, Ito M, Takeuchi N, Koyanagi M, Ogino S (2010). Japanese cedar pollinosis impact on work productivity, quality of life, and symptoms 2008 vs. 2009. Jpn J Rhinol.

[CR18] Ministry of Health, Labour and Welfare of Japan (2011) General survey on part-time workers. http://www.mhlw.go.jp/toukei/list/132-23e.html. Accessed 15 Feb 2016

[CR19] Ministry of Health, Labour and Welfare of Japan (2012) Basic survey on wage structure. http://www.mhlw.go.jp/toukei/itiran/roudou/chingin/kouzou/z2012/index.html. Accessed 15 Feb 2016

[CR20] National Cancer Center, Japan (2001) Center for Cancer Control and Information Services. http://ganjoho.jp/public/. Accessed 15 Feb 2016

[CR21] National Cancer Institute, USA (2014) Surveillance, epidemiology, and end results program. http://seer.cancer.gov/statistics/summaries.html. Accessed 15 Feb 2016

[CR22] Okamoto T, Hashimoto K, Ohashi M, Nakachi T, Ishii A, Miyano S (2004). Survey on health-related quality of life (HRQOL) and cost-effectiveness for rehabiltation hospital inpatients by EuroQOL. Jpn J Rehabil Med.

[CR23] Pauly MV, Nicholson S, Polsky D, Berger ML, Sharda C (2008). Valuing reductions in on-the-job illness: ‘presenteeism’ from managerial and economic perspectives. Health Econ.

[CR24] Reilly MC, Zbrozek AS, Dukes EM (1993). The validity and reproducibility of a work productivity and activity impairment instrument. Pharmacoeconomics.

[CR25] Schultz AB, Edington DW (2007). Employee health and presenteeism: a systematic review. J Occup Rehabil.

[CR26] Schultz AB, Chen CY, Edington DW (2009). The cost and impact of health conditions on presenteeism to employers: a review of the literature. Pharmacoeconomics.

[CR27] Shimozuma K, Sonoo H, Ichihara K, Kurebayashi J, Miyake K, Yoshikawa K, Ota K (1994). Analysis of factors associated with quality of life in breast cancer patients after surgery. Breast Cancer.

[CR28] Tachi T, Teramachi H, Tanaka K, Asano S, Osawa T, Kawashima A, Yasuda M, Mizui T, Nakada T, Noguchi Y, Tsuchiya T, Goto C (2015). The impact of outpatient chemotherapy-related adverse events on the quality of life of breast cancer patients. PLoS One.

[CR29] Taira N, Iwata H, Hasegawa Y, Sakai T, Higaki K, Kihara K, Yamaguchi T, Ohsumi S, Shimozuma K, Ohashi Y (2014). Health-related quality of life and psychological distress during neoadjuvant endocrine therapy with letrozole to determine endocrine responsiveness in postmenopausal breast cancer. Breast Cancer Res Treat.

[CR30] Tanaka K, Tachi T, Asano S, Osawa T, Kawashima A, Hori A, Yasuda M, Mizui T, Nakada T, Tsuchiya T, Teramachi H, Goto C (2015). Impact of outpatient chemotherapy-related adverse effect on daily life and work productivity in breast cancer patients. Jpn J Pharm Health Care Sci.

[CR31] The Japanese Breast Cancer Society (2012) Annual data on breast cancer. http://www.jbcs.gr.jp/SiteMap/sitemap.html. Accessed 15 Feb 2016

[CR32] Wada K, Moriyama M, Narai R, Tahara H, Kakuma R, Satoh T, Aizawa Y (2007). The Effect of Chronic Health Conditions on Work Performance in Japanese Companies. J Occup Health.

[CR33] Wada K, Arakida M, Watanabe R, Negishi M, Sato J, Tsutsumi A (2013). The economic impact of loss of performance due to absenteeism and presenteeism caused by depressive symptoms and comorbid health conditions among Japanese workers. Ind Health.

[CR34] Yamashita M, Arakida M (2006). Concept Analysis of Presenteeism and Its Possible Applications in Japanese Occupational Health. J Occup Health.

